# 2136: Gut microbiome alterations in children undergoing hematopoietic stem cell transplantation

**DOI:** 10.1017/cts.2017.204

**Published:** 2018-05-10

**Authors:** Muna Qayed, Dalia Arafat, Samridhi Banskota, John Horan, Edmund Waller, Gregory Gibson

## Abstract

OBJECTIVES/SPECIFIC AIMS: Aim 1: To compare microbiome diversity among patients who develop BSI post hematopoietic stem cell transplantation (HSCT) and patients without BSI. Aim 2: To compare microbiome diversity among patients who develop moderate to severe acute GVHD post HSCT and patients without GVHD. Aim 3: To describe alterations in specific bacterial orders (Enterobacteriaceae, Clostridia, and Lactobacillales) in pediatric patients undergoing HSCT. METHODS/STUDY POPULATION: Next-generation sequencing of the hypervariable V3 region of the 16s rRNA gene isolated from stool specimens collected at baseline (start of preparative regimen to transplant day), early (day 7–14 post HSCT), and late (day 21–28 post HSCT) from 46 children was performed. Microbiome diversity was assessed by the Shannon index as well as UniFrac analysis, and compared among patients with and without GVHD/BSI. Baseline bacterial diversity was compared among patients by primary diagnosis, race, timing of antibiotic introduction and method of supplemental nutrition. RESULTS/ANTICIPATED RESULTS: Median age was 9 years (range 0.5–19.2 years). There were 36 patients with hematologic malignancies. Patients with nonmalignant disease had a characteristic pattern of microbiome diversity (and microbiota order distribution) at baseline that persisted throughout the first month of transplant (*p*=0.004). Over all patients, there was an early and persistent drop in microbiome diversity throughout the transplant course. Early introduction of broad spectrum antibiotics (prior to transplant day) negatively impacted microbiome diversity (*p*=0.02). There was no difference in microbiome diversity among patients who developed BSI, when compared with patients without BSI. Similarly, we did not find an association between microbiome diversity and the development of moderate to severe acute GVHD. Ongoing analysis is examining the individual variations in microbiome at the species level, and their relation to transplant characteristics and clinical outcomes. DISCUSSION/SIGNIFICANCE OF IMPACT: In our analysis, microbiome diversity decreased during HSCT, but in contrast to published data, mainly in adult HSCT populations, we found no association between gut microbiome diversity and GVHD or BSI. There are ongoing clinical trials in children and adults using probiotics in HSCT with the aim of decreasing GVHD. Further analysis of our data at the species level may further inform the relationship between gut microbiome alterations and HSCT complications in children and guide clinical interventions.

